# Predictors of self-perceived cultural responsiveness in entry-level physiotherapy students in Australia and Aotearoa New Zealand

**DOI:** 10.1186/s12909-019-1487-0

**Published:** 2019-02-13

**Authors:** Maxine Te, Felicity Blackstock, Caroline Fryer, Peter Gardner, Louise Geary, Suzanne Kuys, Kerstin McPherson, Irmina Nahon, Clarice Tang, Lynne Taylor, Gisela Van Kessel, Kelly van der Zwan, Lucy Chipchase

**Affiliations:** 1 School of Science and Health, Western Sydney University, Penrith, NSW 2751 Australia; 2 School of Health Sciences, University of South Australia, Adelaide, SA Australia; 3 School of Physiotherapy and Exercise Science, Curtin University, Bently, WA Australia; 4 School of Allied Health, La Trobe University, Melbourne, VIC Australia; 5 School of Physiotherapy, Australian Catholic University, Brisbane, QLD Australia; 6 School of Community Health, Charles Sturt University, Bathurst, NSW Australia; 7 Faculty of Health, University of Canberra, ACT Bruce, Australia; 8 School of Clinical Sciences, Auckland University of Technology, Auckland, New Zealand; 9 Faculty of Health Sciences and Medicine, Bond University, Gold Coast, QLD Australia

**Keywords:** Cultural responsiveness, Physiotherapy students

## Abstract

**Background:**

Ensuring physiotherapy students are well prepared to work safely and effectively in culturally diverse societies upon graduation is vital. Therefore, determining whether physiotherapy programs are effectively developing the cultural responsiveness of students is essential. This study aimed to evaluate the level of self-perceived cultural responsiveness of entry level physiotherapy students during their training, and explore the factors that might be associated with these levels.

**Methods:**

A cross sectional study of physiotherapy students from nine universities across Australia and Aotearoa New Zealand was conducted using an online self-administered questionnaire containing three parts: The Cultural Competence Assessment tool, Altemeyer’s Dogmatism scale, and the Marlowe-Crowne social desirability scale- short form. Demographic data relating to university, program, and level of study were also collected. Data was analysed using one-way ANOVA, t-tests and multiple regression analysis.

**Results:**

A total of 817 (19% response rate) students participated in this study. Overall, students had a moderate level of self-perceived cultural responsiveness (Mean (SD) = 5.15 (0.67)). Fewer number of weeks of clinical placement attended, lower levels of dogmatism, and greater social desirability were related to greater self-perceived cultural responsiveness. Additionally, fourth year undergraduate students perceived themselves to be less culturally responsive than first and second year students (*p* < 0.05).

**Conclusions:**

These results provide educators with knowledge about the level of self-perceived cultural responsiveness in physiotherapy students, and the factors that may need to be assessed and addressed to support the development of culturally responsive practice.

## Background

Culturally responsive physiotherapy practice is recognised as a vital component of service provision that impacts positively on health outcomes for Indigenous and culturally and linguistically diverse (CALD) communities [1, 2]. Cultural responsiveness refers to the capacity of healthcare professionals or organisations to deliver care that is safe, respectful, and relevant to the health beliefs, practices and cultural and linguistic needs of culturally diverse patient populations [3–5]. Evidence of health disparities experienced by people from Indigenous and CALD communities underpins the need for ensuring culturally responsive practice in all health professions including physiotherapy [6, 7]. As the population of Australia and Aotearoa New Zealand becomes increasingly culturally diverse, physiotherapists must be able to provide culturally responsive care [8–10].

Developing personal cultural responsiveness is a learning process that is ongoing and dynamic [5, 11, 12]. Cultural responsiveness is not an end state, but an “ongoing contextual, developmental and experiential process of personal growth” [12]. While cultural responsiveness is a lifelong journey, there is agreement that aspects of cultural responsiveness should be addressed early in the education of healthcare professionals [13–15]. To be able to design efficacious health professional curricula that supports the development of behaviours, attitudes, and interpersonal interactions that are culturally responsive, educators need to understand the baseline level of cultural responsiveness of healthcare students and the factors that influence cultural responsiveness.

There is a paucity of published research evaluating cultural responsiveness in physiotherapy students. While there are a handful of intervention studies, there have only been two published studies which assess cultural responsiveness throughout the curriculum. Using a modified version of the Self-Assessment of Cultural Competence questionnaire, Doherty et al. [16] found that self-reported cultural responsiveness differed between the year levels, with second year students reporting lower levels than first and third year students. Similarly, Oluwole-Sangoseni & Jenkins-Unterberg [17] found a difference among first, third and sixth year students, although only cultural awareness and sensitivity was assessed. Overall, the results were based on small samples at single tertiary institutions in the United States and was predominately Caucasian females, limiting generalisability of the study results. Further, the results were self-reported perceptions of cultural responsiveness which may have been influenced by social desirability bias [18]. Social desirability refers to an individual’s need for social approval or acceptance, and the belief that this can be attained by adopting socially acceptable behaviours [19–21]. Therefore, to appropriately examine cultural responsiveness using self-reported measures, a measure of social desirability should be concurrently completed, and analyses should include these data as a covariant.

In understanding students’ learning to develop cultural responsiveness, factors that potentially influence development should be considered. To date, no literature has examined factors associated with cultural responsiveness in physiotherapy students. In other healthcare disciplines, students who are female, with greater empathy, self-efficacy and who have a lived experience with CALD communities (including speaking multiple languages), and prior training in cultural responsiveness, have been reported more likely to score higher on self-reported cultural responsiveness measures [22–27]. However, many of these factors have also been reported to not be significant predictors of students’ cultural responsiveness in other studies [28–30]. To date, investigated predictors have been mostly limited to gender, age, ethnicity, exposure to CALD communities, academic level, socioeconomic status and prior training. There has been limited evaluation of the influence of personality traits. In particular, a lack of open-mindedness (dogmatism) is thought to perpetuate negative attitudes and prejudice towards different cultures, and impede the development of cultural responsiveness [31–34]. However, research assessing whether dogmatism is a predictor of cultural responsiveness is lacking. Understanding how dogmatism relates to the level of cultural responsiveness may help discern whether dogmatism needs to be addressed as a component of education to foster cultural responsiveness in physiotherapy students. Therefore, the purpose of this study was two-fold. First, the study aimed to assess the level of self-perceived cultural responsiveness in physiotherapy students in Australia and Aotearoa New Zealand, and to explore whether this differed between year levels. Second, the study also aimed to identify predictors of self-perceived cultural responsiveness, including participant demographics, prior training related to culture or cultural responsiveness, living in a culturally diverse area, number of weeks of clinical placement attended, dogmatism, and social desirability.

## Methods

### Design

This study used a descriptive, cross-sectional design. A self-administered web-based questionnaire was used to collect data from physiotherapy students enrolled in one of nine entry-level physiotherapy programs in Australia or Aotearoa New Zealand. These universities were selected as collaborating research partners, as they offered different program types (bachelor, bachelor/masters combined, graduate entry masters (GEM) or a masters extended), included a range of full fee paying enrolments and government supported financial enrolments, and were spread across different geographical locations (metropolitan and regional) in Australia and Aotearoa New Zealand. The study protocol was approved by Western Sydney University Human Ethics Committee (Approval No. H11967), and was also reviewed and approved by each partner university’s human research ethics committee.

### Data collection procedure

Data collection was conducted between May and November, 2017. Prior to data collection, the researcher at each university provided all participants with full disclosure of their rights, the nature, benefits and risks of the study. This researcher then coordinated a time in a teaching session to provide 20 to 25 min for data collection. During this teaching session, students were provided with a link to the web-based questionnaire. Where this was not feasible due to logistical issues (e.g. students on clinical placement), students were provided with a link via email to complete in their own time. Information about the study was provided at the beginning of the web-based questionnaire, and consent to participate was obtained on the first page of the web-based questionnaire through a check box agreement. The web-based questionnaire consisted of a demographic section and three reliable and valid instruments used previously in the literature to measure cultural responsiveness, dogmatism and social desirability.

### Demographic section

Questions included age in years, gender, postcode, self-identified ethnoculture, and religious affiliation. Participants were also asked about their year level of study, the type of program in which they were enrolled (bachelor, bachelor/masters combined, graduate entry masters or a masters extended) the number of weeks of placement they had attended, if they had prior education or training related to culture or cultural responsiveness, and whether they spoke a language other than English. In Australia and Aotearoa New Zealand, bachelor degrees in physiotherapy are four years, bachelor/masters combined degrees are usually four or five years, graduate entry masters degrees are two years, and masters extended degrees are usually two or three years (or six semesters). A masters extended degree (Doctor of Physiotherapy) is equivalent to a professional doctorate in the United States.

Content validity for this section of the questionnaire was ensured by including questions and answer options based on published work in cultural responsiveness or based on the census data collection in Australia and Aotearoa New Zealand [9, 23, 35, 36]. For example, questions related to demographic variables such as age, gender, level of study, self-identified ethnoculture, type of program or previous cultural training were either adapted from published studies assessing self-perceived cultural responsiveness or constructed based on the literature defining important factors associated with cultural responsiveness. Questions related to ancestry, religious affiliation and spoken language were worded similarly to the Australian 2016 census or Aotearoa New Zealand 2013 census. Additionally, to ensure face validity, a draft of this section was provided to the research team for feedback regarding the content and structure of the questions [37].

### Cultural competence assessment tool

The Cultural Competence Assessment (CCA) [38] was used to assess self-perceived levels of cultural responsiveness. The CCA is a 25 item Likert scale questionnaire with two subscales: Cultural Awareness and Sensitivity (CAS), and Culturally Competent Behaviours (CCB). For an overall CCA score, the average of all 25 items was calculated to provide a score from 1 to 7 [39–41]. The CCA assesses self-report of behaviour rather than self-efficacy for performing a behaviour, and provides a measure of cultural responsiveness that does not emphasise knowledge about specific cultural groups [38].

The CAS subscale measures awareness (knowledge) and sensitivity (attitudes) and consists of 11 items with a 7- point Likert type scale ranging from 1 (Strongly Disagree) to 7 (Strongly Agree), with four items reversed scored. The CCB subscale measures the frequency of culturally responsive behaviours with 14 items and a 7-point Likert type scale ranging from 1 (Never) to 7 (Always). Scores for all items on each subscale are summed and divided by the number of items to provide a score from 1 to 7. Higher scores mean higher levels of overall cultural responsiveness, cultural sensitivity or culturally responsive behaviours demonstrated. Mean scores of 4 indicate moderate levels cultural responsiveness. Mean scores of 5 indicate moderately high levels of cultural responsiveness. Mean scores approaching the range of 6–7 indicate high levels of cultural responsiveness [39, 40]. Internal consistency has been reported as high (Cronbach’s α > 0.80) with validity (content, construct and face) and test-retest reliability established [38, 42, 43].

### Altemeyer’s dogmatism scale

Altemeyer’s dogmatism (DOG) scale was used to assess participants’ level of dogmatism, defined as an unjustified and unchangeable certainty in one’s beliefs [44]. The DOG scale asks respondents to think about the certainty with which they hold their beliefs, their views about maintaining an open belief system, and the likelihood that their beliefs will change in the future [44]. The DOG scale consists of 20 items with a 5-point Likert type scale ranging from 1 (strongly agree) to 5 (strongly disagree). Ten items are reversed scored to avoid response set biases. All items are summed up to calculate the total DOG score. Scores range from 20 to 100, with higher scores indicating greater levels of dogmatism. Internal consistency of the DOG scale has been reported as high (Cronbach’s α > 0.88) and construct validity has been established [44–47].

### Marlowe Crowne social desirability scale – Short form C

The Marlowe Crowne-Social Desirability Scale – Short Form C (MCSD (Form C)) was used to assess participants’ social desirability [18, 48]. The MCSD (Form C) consists of 13 items with a true/false response format. Seven items are reversed scored to avoid response set biases. Scores range from 0 to 13. Higher scores indicate that the participant is more likely to respond in a manner that is considered socially desirable [21, 49]. Internal consistency reliability of the MCSD (Form C) has been reported with a Cronbach’s α ranging from 0.62 to 0.89, and construct validity has been established [50–52].

### Statistical analysis

Demographic characteristics of respondents were analysed using descriptive statistics. Means and standard deviations were reported for cultural responsiveness scores. Associations between academic year levels were analysed based on the type of program (bachelor, graduate entry masters and masters extended) using one-way ANOVAs or independent t-tests, with post hoc tests (Tukey), where appropriate. Differences between universities were not assessed due to political sensitivities and differences in the sample sizes between participating institutions.

To identify predictors of cultural responsiveness, three separate simultaneous multiple linear regression analyses using the general linear model procedure, were conducted for the total CCA score, and the CAS and CCB subscale scores. Predictors are independent variables that are linked or associated with a particular outcome such as the level of cultural responsiveness [53]. Ten independent variables were entered in each model: age, gender, number of weeks of clinical placement attended, prior education related to culture or cultural responsiveness, speaks another language other than English, self-identified ethnoculture, religious affiliation, lives in culturally diverse area, dogmatism score, and social desirability score. These predictors were chosen based on the cultural responsiveness literature, and prior research in other health disciplines [18, 23, 34, 54].

Prior to conducting the analyses, the statistical assumptions for regression analyses were tested. All assumptions were met, and data did not have to be adjusted (i.e. linearity, homoscedasticity and normality of residuals, and multicollinearity were within acceptable limits). Statistical software (Statistical Package for the Social Sciences (SPSS) version 24, IBM Corp., Armonk, NY, USA) was used to perform all the data analysis at a 0.05 level of significance.

## Results

A total of 817 (19% response rate) physiotherapy students from the nine universities in Australia and Aotearoa New Zealand participated. Eighty-five per cent of the responses were undergraduate students, 7% were GEM students, while 6% were enrolled in a masters extended program. Characteristics of the participants are summarised in Table 1.

**Table 1:** Demographic characteristics of the respondents

Demographic Characteristics		Number of students	Percentage (%)
Country of Residence	Australia	717	87
	Aotearoa New Zealand	100	12
Gender^a^	Male	281	34
	Female	529	64
Age (mean years ± SD)		22.6 ± 4.90	
Cohort	1st year undergraduate	193	23
	2nd year undergraduate	193	23
	3rd year undergraduate	171	21
	4th year undergraduate	150	18
	1st year GEM	45	5
	2nd year GEM	14	2
	1st year masters extended	28	3
	2nd year masters extended	23	3
Weeks of clinical placement (mean ± SD)		14.08 ± 10.73	
Prior education or training related to cultural responsiveness^a^	Yes	106	13
	No	704	85
Speaks another language other than English at home	Yes	256	31
	No	561	68
Self-identified Ethnoculture	Indigenous^b^	20	2
	Australian	448	54
	New Zealander	57	7
	Aus/NZ mixed with another ethnoculture	103	13
	Non Aus/NZ	141	17
Religion	No religion	337	41
	Christianity (all denominations)	381	46
	Buddhism	39	5
	Islam	19	2
	Hinduism	23	3
	Other Religions	14	1

^a^Percentages may not add to 100 due to missing data. SD, standard deviation; GEM, graduate entry masters; Aus, Australia; NZ, Aotearoa New Zealand. ^b^NZ Maori and Aboriginal and Torres Strait Islander

### Level of self-perceived cultural responsiveness

The cultural responsiveness mean score was 5.15 ± 0.67 (range = 2.42–6.73), indicating a moderately high level of self-perceived cultural responsiveness among the participants. Responses on the CAS subscale showed a moderately high level of cultural sensitivity and awareness (5.77 ± 0.49, range = 3.27–7.00). Analysis of the CCB subscale showed a moderate level of culturally competent behaviours (4.53 ± 1.11, range = 0–7).

### Self-perceived cultural responsiveness and year levels

There were significant differences between undergraduate year levels for self-perceived cultural responsiveness (overall CCA score) (F[3706] = 4.60, *p* = 0.003) (Table 2). Tukey’s post hoc comparison revealed that fourth year students had lower self-perceived cultural responsiveness when compared to first year (*p* = 0.004) and second year students (*p* = 0.023). There was no statistically significant difference between fourth year and third year students (*p* = 0.46), and between first, second and third year students (all *p* > 0.19).

**Table 2:** Means and standard deviations (SD) for CAS, CCB and total CCA score for each year level

Program Type	Year level	CAS (mean ± SD)	CCB (mean ± SD)	Total CCA (mean ± SD)
Undergraduate	1st year	5.68 ± 0.50^a^	4.80 ± 1.08^bc^	5.24 ± 0.67^e^
	2nd year	5.82 ± 0.50^a^	4.57 ± 1.19^d^	5.25 ± 0.70^f^
	3rd year	5.81 ± 0.44	4.39 ± 1.02^c^	5.09 ± 0.60
	4th year	5.75 ± 0.47	4.23 ± 1.08^bd^	4.94 ± 0.60^ef^
GEM	1st year	5.68 ± 0.48	4.42 ± 1.04	5.03 ± 0.64
	2nd year	5.59 ± 0.67	4.39 ± 0.80	4.96 ± 0.57
Masters extended	1st year	6.01 ± 0.51	5.14 ± 1.00	5.55 ± 0.70
	2nd year	5.99 ± 0.45	4.72 ± 0.98	5.29 ± 0.62

CAS, Cultural Awareness and Sensitivity; CCB, Cultural Competent Behaviours, CCA, Cultural Competence Assessment; GEM, graduate entry masters; SD, standard deviations; ^a^CAS scores 1st year vs 2nd year – *p* < 0.05; ^b^CCB scores 1st year vs 4th year – *p* < 0.05; ^c^CCB scores 1st year vs 3rd year – *p* < 0.05; ^d^CCB scores 2nd year vs 4th year – *p* < 0.05; ^e^CCA scores 1st year vs 4th year; ^f^CCA scores 2nd year vs 4th year

Analysis of the CAS subscale scores revealed a significant difference between undergraduate year levels for cultural awareness and sensitivity (F[3706] = 3.46, *p* = 0.016). Post hoc comparisons revealed that first year students had lower cultural awareness and sensitivity than second year students (*p* = 0.017), but there were no significant differences between all other year level comparisons (all *p* > 0.47).

Analysis of the CCB subscale scores showed significant differences between undergraduate year levels for culturally responsive behaviours (F[3706] = 8.361, *p* < 0.001). Post hoc comparisons revealed that fourth year students perceived they demonstrated less culturally responsive behaviours than first (p < 0.001) and second year students (p = 0.02). Third year students also perceived they demonstrated less culturally responsive behaviours than first year students (*p* = 0.003). There were no significant differences between all other year level comparisons (all *p* > 0.18). There were no significant differences between year levels in the GEM and masters extended programs for self-perceived levels of cultural responsiveness, or for the individual subscales measuring cultural awareness and sensitivity, and culturally responsive behaviours (all *p* > 0.13).

### Predictors of Cultural responsiveness

Multiple regression models for overall self-perceived cultural responsiveness, and subscales of cultural awareness and culturally responsive behaviours are presented in Table 3. All three multiple regression models were significant.

**Table 3:** Multiple regression analysis: Predictors of self-perceived cultural responsiveness

	CAS			CCB			Total CCA		
Predictor variable	B	SE B	t	B	SE B	t	B	SE B	t
Gender (reference group: Male)									
Female	0.06	0.04	1.81	−0.03	0.09	−0.32	0.01	0.05	0.36
Age	0.01	0.04	0.33	0.06	0.09	0.65	0.03	0.05	0.65
Speaks a language other than English (reference group: yes)									
No	0.07	0.05	1.52	0.13	0.11	1.13	0.10	0.07	1.49
Prior cultural related education or training (reference group: yes)									
No	−0.06	0.05	−1.24	−0.08	0.12	−0.67	− 0.07	0.07	− 0.99
Living in a culturally diverse area^a^	−0.03	0.04	− 0.91	− 0.05	0.09	− 0.60	− 0.04	0.05	− 0.82
Number of weeks of clinical placement attended	−0.01	0.03	− 0.33	− 0.26	0.08	−3.29*	− 0.14	0.05	−2.87*
Self-identified ethnoculture (reference group: Australian)									
Indigenous (NZ Maori or Aboriginal and Torres Strait Islander)	0.12	0.12	0.99	0.33	0.29	1.14	0.22	0.17	1.31
New Zealander	−0.04	0.06	−0.59	−0.21	0.15	−1.37	−0.12	0.09	−1.36
Non Australian/New Zealander	−0.04	0.05	−0.85	0.15	0.12	1.25	0.05	0.07	0.76
Australian/New Zealander mixed with other ethnoculture	0.01	0.05	0.13	−0.06	0.13	−0.50	−0.03	0.08	−0.38
Religion (reference group: no religion)									
Christianity	0.05	0.04	1.39	0.17	0.09	1.95	0.11	0.05	2.13
Buddhism	−0.42	0.08	−0.51	0.19	0.20	0.97	0.08	0.12	0.64
Islam	−0.13	0.12	−1.09	0.53	0.29	1.85	0.20	0.17	1.18
Hinduism	0.02	0.10	0.22	0.18	0.25	0.71	0.10	0.15	0.68
Other Religion	0.31	0.14	2.31	0.66	0.33	2.00	0.48	0.19	2.49
Social Desirability	−0.005	0.03	−0.14	0.46	0.08	5.74*	0.23	0.05	4.79*
Dogmatism	−0.31	0.04	−8.87*	− 0.43	0.09	−5.04*	− 0.37	0.05	−7.34*

*CAS* Cultural Awareness and Sensitivity, *CCB* Cultural Competence Behaviour, *CCA* Cultural Competency Assessment, *NZ* Aotearoa New Zealand, *B* unstandardized coefficient, *SE B* standard error for unstandardized coefficient, *t* t test statistics

^a^Data based on the percentage of overseas born population from non-English speaking countries living in the local government area or district in which participants live

*p < 0.01

Fewer number of weeks of clinical placement attended, lower levels of dogmatism, and greater social desirability were significant predictors of greater self-perceived cultural responsiveness, with the model for total CCA score (R^2^ = 0.12, adjusted R^2^ = 0.10, F[17,743] = 6.064, *p* < 0.001) accounting for 10% of the variance in total CCA scores.

Lower levels of dogmatism were a significant predictor of greater self-perceived cultural awareness and sensitivity, with the model for CAS subscale score (R^2^ = 0.15, adjusted R^2^ = 0.13, F[17,743] = 7.309, *p* < 0.001) accounting for 13% of the variance in CAS subscale scores.

Fewer number of weeks of clinical placement attended, lower levels of dogmatism, and greater social desirability were significant predictors of greater self-perceived culturally responsive behaviours, with the model for CCB subscale scores (R^2^ = 0.10, adjusted R^2^ = 0.08, F[17,743] = 4.829, p < 0.001) accounting for 8% of the variance in CCB subscale scores.

## Discussion

This is the first study to assess self-perceived cultural responsiveness in physiotherapy students throughout the curriculum in an Australian and Aotearoa New Zealand context. This study is also the first to explore factors associated with levels of self-perceived cultural responsiveness in physiotherapy students. Understanding baseline levels of cultural responsiveness and the factors that influence cultural responsiveness is central to the development of curriculum that aims to support culturally responsive behaviours, skills and attitudes. The results from this study suggest that physiotherapy educators should consider the characteristics of the learners, especially how dogmatism can contribute to the capacity to develop cultural responsiveness, and the implications of social desirability. Additionally, physiotherapy educators need to be aware of how cultural responsiveness can be fostered overtime.

The results of this study suggest that physiotherapy students who are more dogmatic in their thinking have lower self-perceived cultural responsiveness scores. This may be explained by understanding the cognitive processes related to dogmatism. Dogmatism is a personality trait that is marked by a closed-minded cognitive style. This involves the selective processing of information and evidence, and the tendency to minimise or ignore information that contradicts with co-existing beliefs (confirmatory bias) [55, 56]. Culturally responsive practice requires health professionals to be aware and set aside personal biases, and to understand and respect different health beliefs and experiences from their patients’ perspective [54, 57]. In this sense, individuals who are dogmatic would likely process information about different or competing health beliefs and practices in a biased or dismissive manner.

Previous literature has also demonstrated that dogmatism is associated with negative attitudes and behaviours towards people from different cultural backgrounds. For example, dogmatic nursing staff displayed more negative attitudes or viewed culturally diverse patient groups as more annoying and superstitious than those who were less dogmatic [33]. Additionally, dogmatic students are less willing to listen, and have lower receptivity and tolerance towards teaching instructors who were from CALD communities [58, 59]. These attitudes and behaviours are contrary to the personal attributes that are considered essential for developing culturally responsive practice [34, 54, 60]. Therefore, dogmatism may impede the capacity of students to learn and develop cultural responsiveness. Educators should assess dogmatism to identify at risk students, and design educational interventions that aim to facilitate open-mindedness and self-awareness, and dispel biased and prejudiced thinking to support the development of culturally responsive practice.

In this study, students who responded in a manner considered to be more socially desirable perceived themselves to be more culturally responsive. The majority of studies that have measured social desirability have also demonstrated similar results [18]. Being culturally responsive is a skill that is expected in healthcare culture and practice. Students who are more socially desirable are thought to respond in a way that portrays themselves as favourable, and thus providing a self-perceived measure of their desired performance level rather than actual level [18, 19]. When using self-reported measures to assess cultural responsiveness, social desirability should be assessed to determine the validity of responses [18, 21, 61]. Educators should also consider the implications of social desirability responding on learning. That is, it may be important for educators to have discussions in this area to promote self-awareness in students to openly acknowledge their limitations, and to facilitate self-reflection on skills and behaviours.

Alternatively, greater social desirability and higher self-perceived cultural responsiveness may also be explained by viewing social desirability as a personality trait [62, 63]. Social desirability is associated with personality traits of agreeableness, conscientiousness, emotional stability, greater emotional intelligence, and honest-humility [64–66]. In this sense, social desirability may be indicative of social cognitive skills. That is, individuals are cognisant of the standards of their society or group, are aware of the reputation they hold, and conscious to how they should present themselves in the society or group [67]. As such, physiotherapy students who are more socially desirable may engage in behaviours based on expectations of their roles to provide quality care to their patients. In this view, social desirability may have implications on how educators address this area from a social-cognitive and a professional standpoint. However, social desirability as a personality trait is also influenced by multiple cultural and personality variables [66–68]. More research is required to understand this perspective, how it relates to culturally responsive practice and whether there is a role within curriculum to explore social desirability for learning culturally responsible practice.

The findings in this study also indicate that physiotherapy students with greater clinical experience had lower levels of self-perceived cultural responsiveness than those that did not. While counter-intuitive to what might be expected, these findings have also been observed in other studies [69–72]. On the surface, these results suggest that the curricula in clinical education may not be adequately fostering the development of culturally responsive practice. However, it is also possible that with increasing education and clinical experience, students feel less culturally responsive as they learn more about diversity, and begin to see what they do not know about delivering care to people from CALD communities. Additionally, these results could also represent the increasing ability of students over the duration of their training to effectively self-reflect on their abilities. Understanding how cultural responsiveness is integrated and addressed within the classroom and clinical curricula may help further explain why self-perceived cultural responsiveness decreases overtime. Longitudinal studies assessing cultural responsiveness of students as they progress through the curriculum could also provide better insight into the development of cultural responsiveness overtime.

### Limitations

The findings of this study need to be considered in light of the following limitations. Despite collecting data from 817 students, the overall response rate was 19%. Therefore, there is the risk of non-response bias [73]. However, the sample population included students studying at different universities across different geographical locations in Australia and Aotearoa New Zealand, thereby providing a representative sample of physiotherapy students across the geographic region. Also, demographic data for gender proportions reflect recent studies in Australian physiotherapy universities and the current workforce data [74–77]. In addition, self-reported questionnaires, such as the CCA provide information about perceived abilities, which are often only low or moderately correlated with actual level of performance [78]. As such, self-reported tools may be influenced by social desirability, and the ability of students to accurately self-reflect on their own skills [78, 79]. However, there is little consensus on the most appropriate assessment method and this study attempted to examine the influence of social desirability bias on the cultural responsiveness measure. Also, a vast majority of research relies on self-reported questionnaires, and there are currently no valid and reliable observational measures available to assess cultural responsiveness [18, 79]. Thus, future research should consider developing and validating observational measures to assess cultural responsiveness.

## Conclusion

This study is the first to assess and explore the factors associated with Australian and Aotearoa New Zealand physiotherapy students’ self-perceived cultural responsiveness. The results indicate that higher dogmatism was related to lower levels of self-perceived cultural responsiveness, and higher social desirability was related to higher levels of self-perceived cultural responsiveness. Additionally, students with more clinical experience and final year undergraduate students perceived themselves to be less culturally responsive. Overall, these results provide educators with knowledge about the level of perceived cultural responsiveness in physiotherapy students, and the factors that may need to be assessed and addressed to support the development of culturally responsive practice.

## Abbreviations

CALD: culturally and linguistically diverse
CAS: Cultural awareness and sensitivity
CCA: Cultural competence Assessment
CCB: Cultural competency behaviours
DOG: Altemeyer’s dogmatism scale
GEM: graduate entry masters
MCSD: Marlowe crowne-social desirability scale

## References

[1] Ratima M, Waetford C, Wikaire E (2006). Cultural competence for physiotherapists: reducing inequalities in health between Maori and non-Maori. N Z J Physiother.

[2] Brady B, Veljanova I, Chipchase L (2016). Culturally informed practice and physiotherapy. J Physiother.

[3] Babacan H, Gill GK (2012). Developing a cultural responsiveness framework in healthcare systems: an Australian example. Divers Equal Health Care.

[4] Department of Health. Cultural responsiveness framework: Guidlines for Victoria health services. 2009. https://www2.health.vic.gov.au/about/populations/cald-health.

[5] Muñoz JP (2007). Culturally responsive caring in occupational therapy. Occup Ther Int.

[6] Betancourt JR, Green AR, Carrillo JE, Owusu Ananeh-Firempong I (2003). Defining cultural competence: a practical framework for addressing racial/ethnic disparities in health and health care. Public Health Rep.

[7] Kirmayer LJ (2012). Rethinking cultural competence. Transcult Psychiatry.

[8] Physiotherapy Board of Australia, Physiotherapy Board of New Zealand. Physiotherapy Practice Thresholds in Australia and Aotearoa New Zealand. 2015. https://physiocouncil.com.au/wp-content/uploads/2017/10/Physiotherapy-Board-Physiotherapy-practice-thresholds-in-Australia-and-Aotearoa-New-Zealand.pdf.

[9] Statistics New Zealand. Census QuickStats about culture and identify. 2013:2014 http://archive.stats.govt.nz/Census/2013-census/profile-and-summaryreports/quickstats-culture-identity.aspx. Accessed 6 Aug 2018.

[10] Australian Bureau of Statistics. Census of Population and Housing: Reflecting Australia - Stories from the Census, 2016, cat no. 2071.0. Canberra: ABS. 2017. http://www.abs.gov.au/ausstats/abs@.nsf/Lookup/by%20Subject/2071.0~2016~Main%20Features~Cultural%20Diversity%20Article~60. Accessed 12 Sept 2018.

[11] Beagan BL (2015). Approaches to culture and diversity: a critical synthesis of occupational therapy literature. Can J Occup Ther.

[12] Suarez-Balcazar Y, Rodakowski J (2007). Becoming a culturally competent occupational therapy practitioner: practical ways to increase cultural competence. OT Pract.

[13] Betancourt JR (2006). Cultural competence and medical education: many names, many perspectives, one goal. Acad Med.

[14] Seeleman C, Suurmond J, Stronks K (2009). Cultural competence: a conceptual framework for teaching and learning. Med Educ.

[15] Sit A, Mak AS, Neill JT (2017). Does cross-cultural training in tertiary education enhance cross-cultural adjustment? A systematic review. Int J Intercult Relat.

[16] Doherty D, Maher SF, Ivanikiw C, Hales M, Lebiecki T, Wren PA (2017). Perceptions of cultural competency in doctor of physical therapy students introduction. J Cult Divers.

[17] Oluwole-Sangoseni O, Cultural Awareness J-UM (2017). Sensitivity of students in a physical therapy program–a pilot survey. Internet journal of allied health sciences and. Practice.

[18] Larson KE, Bradshaw CP (2017). Cultural competence and social desirability among practitioners: a systematic review of the literature. Child Youth Serv Rev..

[19] Fisher R, Katz JE (2000). Social-desirability bias and the validity of self-reported values. Psychol Mark.

[20] Marlowe D, Crowne DP (1961). Social desirability and response to perceived situational demands. J Consult Psychol.

[21] Van de Mortel TF (2008). Faking it: social desirability response bias in self-report research. Aust J Adv Nurs.

[22] Seo YS, Kwon Y-C (2014). Factors influencing to the cultural competence in nursing students. Journal of Digital Convergence.

[23] Cruz JP, Aguinaldo AN, Estacio JC, Alotaibi A, Arguvanli S, Cayaban ARR, John Cecily HS, Machuca Contreras FA, Hussein A, Idemudia ES (2018). A multicountry perspective on Cultural competence among baccalaureate nursing students. J Nurs Scholarsh.

[24] Dunagan PB, Kimble LP, Gunby SS, Andrews MM (2014). Attitudes of prejudice as a predictor of cultural competence among baccalaureate nursing students. J Nurs Educ.

[25] Constantine MG (2001). Multiculturally-focused counseling supervision. Clin Superv.

[26] Lee A, Khawaja NG (2013). Multicultural training experiences as predictors of psychology Students' Cultural competence. Aust Psychol.

[27] Mirsu-Paun A, Tucker CM, Hardt NS (2012). Medical students' self-evaluations of their patient-centered cultural sensitivity: implications for cultural sensitivity/competence training. J Natl Med Assoc.

[28] Jeffreys MR, Dogan E (2012). Evaluating the influence of cultural competence education on students' transcultural self-efficacy perceptions. J Transcult Nurs.

[29] Lim J, Downie J, Nathan P (2004). Nursing students' self-efficacy in providing transcultural care. Nurse Educ Today.

[30] Kim DH, Kim SE (2013). Cultural competence and factors influencing Cultural competence in nursing students. J Korean Acad Psychiatr Ment Health Nurs.

[31] Bonaparte BH (1979). Ego defensiveness, open-closed mindedness, and nurses' attitude toward culturally different patients. Nurs Res.

[32] Hannigan TP (1990). Traits, attitudes, and skills that are related to intercultural effectiveness and their implications for cross-cultural training: a review of the literature. Int J Intercult Relat.

[33] Ruiz MC (1981). Open-closed mindedness, intolerance of ambiguity and nursing faculty attitudes toward culturally different patients. Nurs Res.

[34] Jenks AC (2011). From "lists of traits" to "open-mindedness": emerging issues in cultural competence education. Cult Med Psychiatry.

[35] Silvestri-Elmore AE, Alpert PT, Kawi J, Feng D (2016). The predictors of cultural competence among new baccalaureate degree nursing graduates: implications for nursing education. J Nurs Educ Pract.

[36] Australian Bureau of Statistics. Census of Population and Housing. Nature and content. Australia, 2016, cat. 2016;2008:0

[37] Bolarinwa OA (2015). Principles and methods of validity and reliability testing of questionnaires used in social and health science researches. Niger Postgrad Med J.

[38] Schim SM, Doorenbos AZ, Miller J, Benkert R (2003). Development of a Cultural competence assessment instrument. J Nurs Meas.

[39] Cicolini G, Della Pelle C, Comparcini D, Tomietto M, Cerratti F, Schim SM, Di Giovanni P, Simonetti V (2015). Cultural competence among Italian nurses: a multicentric survey. J Nurs Scholarsh.

[40] Doorenbos AZ, Morris AM, Haozous EA, Harris H, Flum DR (2016). Assessing Cultural competence among oncology surgeons. J Oncol Pract.

[41] Heitzler ET (2017). Cultural competence of obstetric and neonatal nurses. J Obstet Gynecol Neonatal Nurs.

[42] Doorenbos AZ, Schim SM, Benkert R, Borse NN (2005). Psychometric evaluation of the cultural competence assessment instrument among healthcare providers. Nurs Res.

[43] Schim SM, Doorenbos AZ, Borse NN (2005). Cultural competence among Ontario and Michigan healthcare providers. J Nurs Scholarsh.

[44] Altemeyer B. The authoritarian specter: Harvard University Press; 1996.

[45] Crowson HM (2009). Does the DOG scale measure dogmatism? Another look at construct validity. J Soc Psychol.

[46] Altemeyer B (2002). Dogmatic behavior among students: testing a new measure of dogmatism. J Soc Psychol.

[47] Crowson HM, DeBacker TK, Davis KA (2008). The DOG scale: a valid measure of dogmatism?. J Individ Differ.

[48] Reynolds WM (1982). Development of reliable and valid short forms of the Marlowe-Crowne social desirability scale. J Clin Psychol.

[49] Crowne DP, Marlowe D (1960). A new scale of social desirability independent of psychopathology. J Consult Psychol.

[50] Barger SD (2002). The Marlowe-Crowne affair: short forms, psychometric structure, and social desirability. J Pers Assess.

[51] Loo R, Loewen P (2004). Confirmatory factor analyses of scores from full and short versions of the Marlowe–Crowne social desirability scale. J Appl Soc Psychol.

[52] Loo R, Thorpe K (2000). Confirmatory factor analyses of the full and short versions of the Marlowe-Crowne social desirability scale. J Soc Psychol.

[53] Cohen J, Cohen P, West SG, Aiken LS. Applied multiple regression/correlation analysis for the behavioral sciences: Routledge; 2013.

[54] Henderson S, Horne M, Hills R, Kendall E (2018). Cultural competence in healthcare in the community: a concept analysis. Health Soc Care Community.

[55] Ottati V, Price ED, Wilson C, Sumaktoyo N (2015). When self-perceptions of expertise increase closed-minded cognition: the earned dogmatism effect. J Exp Soc Psychol.

[56] Price E, Ottati V, Wilson C, Kim S (2015). Open-minded cognition. Personal Soc Psychol Bull.

[57] Schim SM, Doorenbos AZ (2010). A three-dimensional model of cultural congruence: framework for intervention. J Soc Work End Life Palliat Care.

[58] Roberts CV, Vinson L (1998). Relationship among willingness to listen, receiver apprehension, communication apprehension, communication competence, and dogmatism. International Journal of Listening.

[59] Bresnahan MI, Kim MS (1993). Predictors of receptivity and resistance toward international teaching assistants. Journal of Asian Pacific Communication.

[60] Schim SM, Doorenbos A, Benkert R, Miller J (2007). Culturally congruent care: putting the puzzle together. J Transcult Nurs.

[61] Lambert CE, Arbuckle SA, Holden RR (2016). The Marlowe–Crowne social desirability scale outperforms the BIDR impression management scale for identifying fakers. J Res Pers.

[62] McCrae RR, Costa PT (1983). Social desirability scales: more substance than style. J Consult Clin Psychol.

[63] Jan-Erik L, Sampo P, Annamari T-H, Jouko L, Markku V (2007). Substance and style in socially desirable responding. J Pers.

[64] Mesmer-Magnus J, Viswesvaran C, Deshpande S, Jacob J (2006). Social desirability: the role of over-claiming, self-esteem, and emotional intelligence. Psychol Test Assess Model.

[65] de Vries RE, Zettler I, Hilbig BE (2014). Rethinking trait conceptions of social desirability scales: impression management as an expression of honesty-humility. Assessment.

[66] Connelly BS, Chang L (2016). A meta-analytic multitrait multirater separation of substance and style in social desirability scales. J Pers.

[67] Lalwani AK, Shavitt S, Johnson T (2006). What is the relation between cultural orientation and socially desirable responding?. J Pers Soc Psychol.

[68] Parmač Kovačić M, Galić Z, Jerneić Ž (2014). Social desirability scales as indicators of self-enhancement and impression management. J Pers Assess.

[69] Alpers RR, Zoucha R (1996). Comparison of cultural competence and cultural confidence of senior nursing students in a private southern university. J Cult Divers.

[70] Ladson GM, Lin JM, Flores A, Magrane D (2006). An assessment of cultural competence of first- and second-year medical students at a historically diverse medical school. Am J Obstet Gynecol.

[71] White-Means S, Zhiyong D, Hufstader M, Brown LT (2009). Cultural competency, race, and skin tone bias among pharmacy, nursing, and medical students: implications for addressing health disparities. Med Care Res Rev.

[72] Rew L, Becker H, Chontichachalalauk J, Lee HY (2014). Cultural diversity among nursing students: reanalysis of the cultural awareness scale. J Nurs Educ.

[73] Sax LJ, Gilmartin SK, Bryant AN (2003). Assessing response rates and nonresponse Bias in web and paper surveys. Res High Educ.

[74] Connaughton J, Gibson W (2016). Physiotherapy Students' attitudes toward psychiatry and mental health: a cross-sectional study. Physiotherapy Canada Physiotherapie Canada.

[75] Edgar S (2015). Identifying the influence of gender on motivation and engagement levels in student physiotherapists. Med Teach.

[76] Knox GM, Snodgrass SJ, Stanton TR, Kelly DH, Vicenzino B, Wand BM, Rivett DA (2017). Physiotherapy students' perceptions and experiences of clinical prediction rules. Physiotherapy.

[77] Physiotherapy Board of Australia. Physiotherapy Board of Australia Registrant data. 2018. https://www.physiotherapyboard.gov.au/about/statistics.aspx. Accessed 7th January 2019.

[78] Davis DA, Mazmanian PE, Fordis M, Van Harrison RR, Thorpe KE, Perrier L (2006). Accuracy of physician self-assessment compared with observed measures of competence: a systematic review. JAMA.

[79] Gozu A, Beach MC, Price EG, Gary TL, Robinson K, Palacio A, Smarth C, Jenckes M, Feuerstein C, Bass EB (2007). Self-administered instruments to measure cultural competence of health professionals: a systematic review. Teach Learn Med.

